# Effect of Fluoride Concentration in Drinking Water on Dental Fluorosis in Southwest Saudi Arabia

**DOI:** 10.3390/ijerph17113914

**Published:** 2020-06-01

**Authors:** Gotam Das, Vineet Tirth, Suraj Arora, Ali Algahtani, Mohammed Kafeel, Ayed Hassan G Alqarni, Priyanka Saluja, Hitesh Vij, Shashit Shetty Bavabeedu, Amit Tirth

**Affiliations:** 1 Department of Prosthodontics, College of Dentistry, King Khalid University, Abha 61411, Saudi Arabia; 2 Mechanical Engineering Department, College of Engineering & Research Center for Advanced Materials Science (RCAMS), King Khalid University, Abha 61411, Saudi Arabia; v.tirth@gmail.com (V.T.); alialgahtani@kku.edu.sa (A.A.); 3 Restorative Dental Sciences, College of Dentistry, King Khalid University, Abha 61411, Saudi Arabia; surajarorasgrd@yahoo.co.in (S.A.); sbavabeedu@kku.edu.sa (S.S.B.); 4 Chemical Engineering Department, College of Engineering, King Khalid University, Abha 61411, Saudi Arabia; mokafeel@kku.edu.sa; 5 Intern, College of Dentistry, King Khalid University, Abha 61411, Saudi Arabia; dent.alqarni@gmail.com; 6 Department of Conservative Dentistry and Endodontics, JCD Dental College, Vidyapeeth, Sirsa 125055, Haryana, India; priyanka.salujaarora@gmail.com; 7 Boston University Henry M. Goldman School of Dental Medicine, Boston, MA 02118, USA; hiteshvij@gmail.com; 8 Department of Public Health Dentistry, Kothiwal Dental College and Research Center, Moradabad, 244001 UP, India; atirth@yahoo.co.in

**Keywords:** fluoride levels, drinking water, dental fluorosis, well water, bottled water, filtered water

## Abstract

This study was intended to evaluate the fluoride concentration in drinking water and its effect on dental fluorosis in Southwest Saudi Arabia. Water samples were gathered rom wells, filtration plants and commercial brands (bottled water) in distinct urban and rural areas of Asir region of the Kingdom of Saudi Arabia (KSA). Overall, 63 water samples were collected from 12 locations and 9 brands of bottled water. ExStik^®^ FL700Fluoridemeter was used in the analysis of water samples for fluoride levels. The total number of screened patients for dental fluorosis, aged between 9 and 50 years, was 1150; among them, 609 were males and 541 were females. Dean’s index criteria were used to examine the patients for dental fluorosis. The results revealed that fluoride levels varied between 0.03 and 3.8 ppm. People who drank well water displayed increased fluoride levels (>0.81 ppm). The prevalence of dental fluorosis was established to be 20.43% among the total number of examined patients. The findings of this study show very mild to moderate dental fluorosis prevail among the patients who consume well water in the Asir region.

## 1. Introduction

Fluorine, which is extensively circulated in the environment, is a highly electronegative ion, so it can easily and quickly make bonds with the positive elements. Fluorine, a natural element, is readily dissolved in water, soil and air, and does not occur on its own in nature but occurs relatively as fluoride [1]. Fluoride is beneficial for humans, however an excess of this ion can produce numerous adverse effects on human health [1,2]. Surface water is usually low in fluoride, with a value lower than 1.5 mg/L, although groundwater can contain an increased level of fluoride depending on geographical situations. Increased fluoride levels in drinking water above 1.5 mg/L causes a higher risk of dental fluorosis, though increasingly greater concentrations can lead to the risk of skeletal fluorosis as well [2].

Controlled community water fluoridation bears a major responsibility in improving oral health by decreasing the burden of tooth decay. The Centers for Disease Control and Prevention has established community drinking water fluoridation as one of the greatest public health accomplishments of the 20th century [3]. It has been discovered in the USA that water fluoridation at an optimum level reduces dental decay by around 20–40%. In England, water fluoridation has been established to decrease tooth caries by 44% in preschool children aged around five years [3,4]. Water and water-based drinks are the chief cause of systemic intake of fluoride; approximately 75% of dietary fluoride ingestion in the population is due to fluoridated water [5]. Several processes of fluoride intake are presented, which are in the form of topical fluoride or systemic fluoride. Of all accessible techniques, adding fluoride to community drinking water is the most inexpensive and one of the safest mechanisms for delivering fluoride [6]. The major supply of systemic fluoride in kids is from water (tap and bottled), carbonated drinks, and juices [7]. Internationally, the immense majority of customers that consist of kids and youngsters are switching their everyday water intake to bottled water, possibly due to the fear of a reduction in the purity of natural water delivery and the occurrence of pollutants such as sand particles or microorganisms [8].

In the Kingdom of Saudi Arabia (KSA), tooth decay is an important public health issue, affecting the primary dentition of around 95%of kids aged 3–7 years, around 91% youngsters aged between 12 and 19 years, and 98% of adults aged between 30 and 45 years [3].Especially, in the eastern province of KSA, the overall frequency of dental caries among children aged between 6 and 9 years and10 and 12 years is 73% and 68%, respectively [3].Water fluoridation is considered as a cost-effective measure for caries-prevention in individuals of moderate to high caries risk. In addition, it has a strong safety profile, with the only side effect being a low risk of very mild fluorosis (~2%), which itself may not essentially be due to the exposure to increasing levels of fluoride alone [2,5,6].A study showed that 75% of public water supplied in the central province cities of Riyadh and Qassim has fluoride levels between 0 and 6 ppm [9].One more study performed in three cities of the western province of Saudi Arabia discovered varying fluoride levels, ranging from0.3 to 2.5 ppm, depending on the water supply source [3]. However, the US Department of Health and human services suggests that the optimal water fluoride level for humans with negligible risk is 0.7 ppm [10].

Dental fluorosis is a developmental interruption of enamel, caused by increased ingestion of high concentrations of fluoride at the time of tooth development, leading to enamel with lower mineral substance and greater porosity [11]. Excessive fluoride intake can lead to dental fluorosis. The World Health Organization (WHO) published in its report that around 70 million people in the world maybe affected with dental fluorosis, of which 60 million would be in India and China alone [12]. The mechanism of dental fluorosis is a replacement of the organic enamel matrix by inorganic material, which results in hypomineralizationin the enamel [13]. Tooth fluorosis in small levels causes distinct alteration in tooth shade. On the contrary, severe forms of tooth fluorosis result in the pitting and staining of the dental enamel [11]. According to the data of tooth fluorosis, the younger children who have resided in the fluoridated region for a long time are vulnerable to dental fluorosis, as fluoride readily targets enamel in the mineralization and developmental process of tooth formation [14].

The review of the literature reveals that it is important to determine the fluoride concentration in drinking water and compare it with the acceptable levels to prevent tooth fluorosis. This study is among a few studies performed in the Asir region of Kingdom of Saudi Arabia to evaluate the fluoride content in drinking water. The current study aims to evaluate fluoride levels in drinking water and the occurrence of dental fluorosis in the Southwest region of KSA. There are no statistics available on the level of fluoride in the drinking water in the Asir region of Saudi Arabia. To fill this gap, this study was accomplished to explore baseline data concerning fluoride levels in drinking water, and to evaluate the dental fluorosis.

## 2. Methods

The approach of water sampling in this cross-sectional study was based on the drinking water sources in the Asir region. The water sources included were well water, filtered water and bottled water in the southern region. A total of 63 water samples were gathered from 12 regions/cities and 9 brands of bottled water in the Asir region of KSA. To overcome any variability across seasons, five water samples were gathered from the wells and the filtration plants and three water samples were taken for bottled water. Aldosari et al. [9] suggested a water collection technique and protocol, and the same was used in this study: water was allowed to run for 3 min, after which the 500 ml polythene bottles used for collection were rinsed three times, and then 300 mL of the sample was collected. Every sample was examined using an ion chromatography system (ExStik^®^ FL700Fluoride Meter, USA), after calibration and according to the manufacturer’s instructions. To ensure consistency in measurements, the system was set at different recognition limits, including 2 ppm, 1 ppm, 0.5 ppm, and the lowest detection limit of 0.01 ppm, and readings were recorded if fluoride was identified in the sample at the selected detection limit. Multiple readings at the same detection limit were carried out and all samples were analyzed within 3 days of collection.

**Figure 1:**
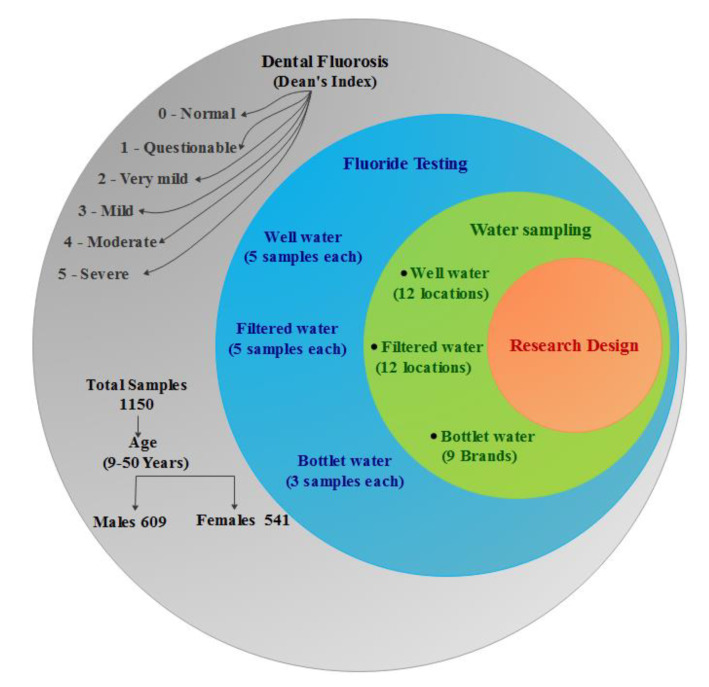
The research design.

This study was carried out between July and December 2019 among the dental in patients visiting the dental college in Abha-Asir, KSA. The patients included those with pre-existing dental issues, as well as those that turned up for routine check-up. The sample also includes the general population of Southwest KSA, who are examined during frequently organized community dental check-up camps. Stratified random sampling was used to find out the sample size. The research design is presented in Figure 1. A total of 1150 patients, aged between 9 and 50 years, were checked, out of which 609 were male and 541 were females. The inclusion criteria for this research were volunteer participation, the age bracket of 9–50 years, and only those subjects with fully erupted primary or permanent teeth were considered. The examination was done by two dentists; they were assisted by two dental assistants for data recording. The Dean’s index was used to grade the severity of fluorosis (0—normal, 1—questionable, 2—very mild, 3—mild, 4—moderate, and 5—severe). All the subjects were asked to brush their teeth under the supervision of a team member. The teeth were dried with cotton pallet and examination was done visually using a dental mirror, explorer, and tongue depressor as suggested by the WHO.

## 3. Results

**Figure 2:**
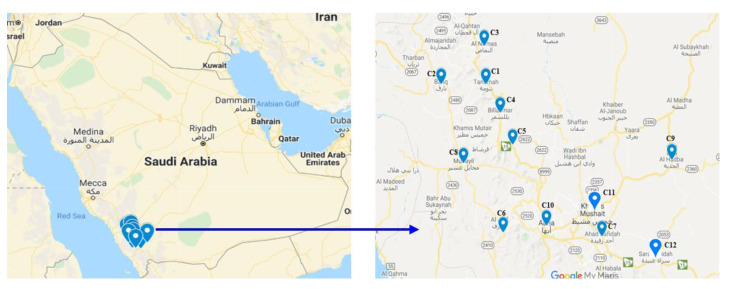
Locations in Southwest region of Kingdom of Saudi Arabia (KSA) from which the water samples were collected.

There are four major sources of drinking water in the Asir region: the water supplied by public works or contracted companies, the well water (in the countryside and remote areas), filtered water available from the filtration plants in all major urban and suburban areas, and commercially bottled water. The water supply to the filtration plants is by public works or contracted companies. The samples taken from the 12 collection points in the southern regions are presented in Figure 2. The codes assigned to the cities/regions with their GPS location is given in Table 1. The fluoride content in well water at he 12 locations, shown in Figure 2, is given in Table 2. The minimum and maximum fluoride content is 0.6 and 3.8 ppm respectively. The mean fluoride across the 12 locations is 1.97 ppm. The standard deviation varies from 0.2 to 1.09. The average of the minimum and the maximum, as well as the mean values, of all the well water samples of the12 locations is estimated as 1.24, 2.78 and 1.97 ppm, respectively. The fluoride content in the filtered water in the 12 cities is given in Table 3. The minimum and maximum fluoride content is 0.03 and 2.55 ppm respectively, and the mean fluoride content across all the cities is 1.05 ppm. The standard deviation is between 0.51 and 1.03. The average of the minimum and the maximum, as well as the mean values, of all the filtered water samples of the 12 locations is estimated as 0.27, 2.24 and 1.05 ppm, respectively. The fluoride content in the commercially bottled water (from three samples) is given in Table 4. The minimum and maximum fluoride content in bottled water is 0.8 and 1.9 ppm respectively, and the mean fluoride content across all the cities is 1.09 ppm. The standard deviation is between 0.06 and 0.12. The average of the minimum and the maximum, as well as the mean values, of all the nine brands of bottled water samples is estimated as 0.91, 1.18 and 1.09 ppm, respectively.

Higher fluoride content is observed in the well water and a few samples of filtered water. Largely, the average fluoride content in the filtered water and the bottled water was within the acceptable limits [10]. The standard deviation in the samples collected from wells and the filtration plants may be due to the variation in the source. The fluoride content is high in the well water in particular.

**Figure 3:**
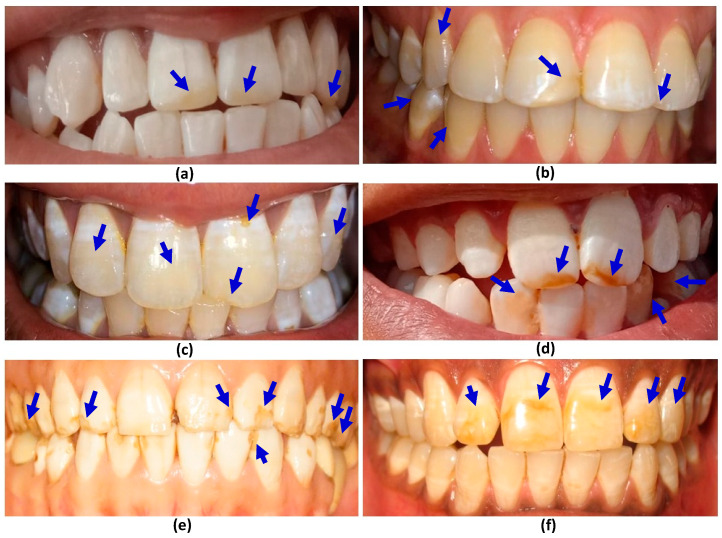
Pictures of teeth, arrows indicate: (**a**,**b**) very mild fluorosis; (**c**,**d**) mild fluorosis; (**e**,**f**) moderate fluorosis.

The dataset for the dental fluorosis detected across a sample of 1150 people in the Asir regionof Southwest KSA is given in Table 5. Dental fluorosis is related to the drinking water consumed by the patients who source it from the well and filtration plants. The total number of people who use well water was 495, about 43% of the sample. People who drink filtered water were 57%. No fluorosis was detected in about 50% of the sample, and about 29% of the sample exhibited questionable symptoms. Fluorosis was detected in the remaining 20.43%of the sample size, classified in four categories, viz. very mild, mild, moderate and severe, as given in Table 5. The occurrence of very mild fluorosis across the total sample was 12.2%, out of which only 3.1% of patients were those who drank filtered water, and 9.1% were people who drank well water. The mild fluorosis was observed in 6.6% of patients, 6.1% in those who drank well water, and only 0.5% in the people who drank filtered water. The occurrence of moderate and severe fluorosis was quite low in the samples. The moderate fluorosis was observed in only 1.3% of the sample, 1% in those who used well water for drinking and 0.3% in those who drank filtered water. The severe fluorosis was detected only in 0.26% of people who drank well water and it was not observed in the people who drank filtered water. In some of the regions, the water supply is there but it is restricted, so the people largely use well water for drinking. The severity of dental fluorosis in subjects who drank well or filtered water is shown by pictures in Figure 3. Figure 3a,b show very mild fluorosis, Figure 3c,d show mild fluorosis, and Figure 3e,f show moderate fluorosis. All types of fluorosis can be distinctly identified in the images. The typical build-up of fluorosis is marked by the blue arrows in the pictures.

## 4. Discussion

Drinkable water consists of desalinated water or a mixture of desalinated water and groundwater. It is provided at low-cost rates to two-thirds of the households in the region. Community water fluoridation is recognized to play an important part in improving oral health by decreasing the prevalence of dental caries [15]. Further, it is a safe and cost-effective mechanism to decrease dental decay way for large-scale populations [16], but when fluoride level increase in drinking water it may cause tooth fluorosis [17]. The present study gives a clue about the fluoride levels in drinking water in the Asir region of KSA and the occurrence of dental fluorosis. 

The Kingdom of Saudi Arabia has two important origins of drinking water: traditional sources, including groundwater (40%) and surface water such as rain; and unconventional sources, including desalinated water (50%) and treated wastewater (10%) [3]. Saudi Arabia depends primarily on desalinated water because of dominant desert demography and the lack of adequate natural water resources [18]. According to the Joint Monitoring Program for Water Supply and Sanitation by WHO and United Nations International Children’s Emergency Fund, it was predictable that the residents of Saudi Arabia would have access to an appropriate source of drinking water, as of 2015 [19]. The southern region obtains desalinated water, as its major source of domestic water, from plants in the Asir region, as in other regions of KSA [20]. This study established that in the Asir region, drinking water is obtained from three main sources: (1) the well (underground) water in the countryside and remote areas; (2) the filtered water obtained from the filtration plants available in urban and suburban areas (the water supply to the filtration plants is from the public supply or the contracted companies); (3) commercial companies, which supply bottled water. The present study gathered samples from all these sources. The mean fluoride level in the well water in 12 regions of the southern region was 1.97 ppm. The mean fluoride level in the filtered water in 12 cities or regions of the southern region was 1.05 ppm. The mean fluoride content in nine commercial bottled water brands was found to be 1.09 ppm.

This study established that, though the fluoride level in the well water in the Asir region was above the recommended level of 0.7–1.2 ppm [10], the well water samples could be studied differently, because the well water is used for drinking and other domestic uses only in the countryside where the municipal water supply is absent. The fluoride level in filtered and bottled water was well within the recommended level. These outcomes are in some contrast with the other results of similar studies performed in other regions of KSA. In the western region of Saudi Arabia, fluoride levels in drinking water were found to be 2.5 ppm in Makkah, 0.8 ppm in Rabagh, and 0.3 ppm in Jeddah [9]. In Riyadh, Hail and Qassim fluoride levels varied greatly, and were found to be 0.00, 2.8, and 6.20 ppm, respectively [21]. The southern region has a large number of origins of groundwater supply, with a mean natural fluoride level of 1.97 ppm. In the western province of KSA, Al-Khateeb et al. [21] found that high water fluoride levels were associated with an increase in dental fluorosis. In contrast, in Riyadh and Qassim, no linear relationship was found between water fluoride levels and caries [22]. However, in the Hail region, high levels of water fluoride were found to be correlated with dental fluorosis [23]. There is some variability in the fluoride levels in drinking water supplied to different areas of KSA, and in different commercially bottled water [4,9,24].One limitation of this study is that only 12 locations of the Asir region are included, and so the results may not be representative of the entire Asir region of KSA. Future studies may include other urban and nonurban cities in. Another shortcoming is that the study did not measure dental caries to explore if any linear relationship exists between fluoride levels and prevalence of dental caries, and thus future studies may be conducted to determine the same. It appears that the KSA government is monitoring the fluoride levels in the water supplied by public works and the contracted companies is safe, as the fluoride content in the filtered water, which is sourced from these sources, was within the recommended range. However, before setting standards for regulating water fluoride levels, it would be significant to establish the daily water intake of the population and evaluate their use of publicly supplied water, in addition to their exposure to other fluoride sources. Therefore, the authors recommend conducting a comprehensive analysis, including all cities in the southern region of KSA, to determine the percentage of the population who use drinking water through the identified sources in this study. The results of the current study showed that the existence of mild fluorosis was the highest among the categories of dental fluorosis. It was found that out of the total number of screened subjects; around 12.81% were affected by very mild fluorosis, 6.9% by mild, 1.3% by moderate and only 0.2% by severe degrees. The results are nearly similar to the findings of a study conducted in the Dammam region of KSA [25]. The dental fluorosis observed in this study is less than the incidence of dental fluorosis (59.72%) among children of Najran, KSA, reported in another study [26]. Hence, the results may not be generalized, as they vary from region to region and need further and wider investigation.

## 5. Conclusions

This study revealed that the fluoride concentration in identified sources of water, in the southwestern region of KSA at 12 locations, was found to be within the recommended levels in filtered and bottled water, but high in well water. Dental fluorosis was also slightly greater in patients who drank well water. The frequency of dental fluorosis among the population was not high (20.43%) in southern region compared with the other regions of KSA. Fluoride levels were up to the mark in different brands of local and imported bottled water. Meeting the fluoride levels of bottled water is important to prevent fluorosis, particularly in regions where bottled water is not available. The study suggests discarding the use of well water for drinking purposes and recommending water from the public supply, contracted companies, filtration plants and bottled water for drinking, to eliminate the build-up of dental fluorosis.

There may be other reasons for the build-up of dental fluorosis, which are not in the scope of this study. Based on the results of this exploratory study, further research on dental fluorosis is recommended at a wider scale to derive baseline data for various regions of KSA, which can set future directions of research. It is also recommended to have studies on other contributing factors, such as dietary habits, for instance, consumption of local tea called Gahwa, juices, milk and its products, and the intake of fluoride-containing oral cleansing agents.

## Figures and Tables

**Table 1:** Codes assigned to the cities/regions, with GPS location, from which well water and filtered water were collected in the Southwest region of KSA.

City/Regions	GPS Location	Code Assigned to Well Water	Code Assigned to Filtered Water
Tanomah	18.92901, 42.17702	C1	F1
Belqarn	18.93053, 41.92956	C2	F2
Al Namas	19.11342, 42.16714	C3	F3
Bellasmar	18.79251, 42.25596	C4	F4
Bellahmar	18.63841, 42.32379	C5	F5
Rigal Alma	18.21253, 42.27384	C6	F6
Ahad Rafidah	18.1952, 42.82051	C7	F7
Muhayil	18.54739, 42.05343	C8	F8
Tareeb	18.56551, 43.20596	C9	F9
Abha	18.24646, 42.51172	C10	F10
KhamisMushayat	18.30933, 42.76623	C11	F11
SaratAbedah	18.09931, 43.11656	C12	F12

**Table 2:** Fluoride content in well water in the 12 cities in Southwest region of KSA.

Water Source	Code	No. of Samples	Fluoride Content (ppm)			Std. Deviation
			Min.	Max.	Mean	
Well Water	C1	5	1.10	3.50	2.44	0.97
	C2	5	1.30	3.80	2.24	0.99
	C3	5	1.05	3.80	2.10	1.09
	C4	5	1.70	3.70	2.34	0.81
	C5	5	1.50	3.20	2.12	0.66
	C6	5	1.30	2.20	2.21	0.71
	C7	5	1.10	1.90	1.69	0.34
	C8	5	1.75	3.10	2.33	0.63
	C9	5	1.15	2.00	1.43	0.35
	C10	5	1.20	2.40	1.78	0.49
	C11	5	1.10	2.60	2.08	0.63
	C12	5	0.60	1.10	0.82	0.20
	Average/mean		1.24	2.78	1.97	

**Table 3:** Fluoride content in filtered water in the 12 cities in Southwest region of KSA.

Water Source	Code	No. of Samples	Fluoride Content (ppm)			Std. Deviation
			Min.	Max.	Mean	
Filtered Water	F1	5	0.40	2.15	1.11	0.65
	F2	5	0.30	2.55	1.02	0.89
	F3	5	0.03	2.45	0.89	1.03
	F4	5	0.05	2.45	1.12	0.86
	F5	5	0.07	2.55	0.98	0.94
	F6	5	0.40	2.05	1.11	0.76
	F7	5	0.08	2.25	1.19	0.80
	F8	5	0.50	1.75	1.05	0.51
	F9	5	0.30	2.05	0.93	0.70
	F10	5	0.30	2.05	0.91	0.68
	F11	5	0.30	2.25	1.11	0.77
	F12	5	0.50	2.35	1.21	0.69
	Average/mean		0.27	2.24	1.05	

**Table 4:** Fluoride content in commercially bottled water consumed in Southwest region of KSA.

Water Source	Code	No. of Samples	Fluoride Content (ppm)			Std. Deviation
			Min.	Max.	Mean	
Bottled Water	B1	3	0.8	1	0.92	0.10
	B2	3	1.2	1.4	1.30	0.10
	B3	3	0.8	1.9	1.83	0.06
	B4	3	0.9	1.1	1.00	0.10
	B5	3	0.9	1	0.93	0.06
	B6	3	1	1.1	1.03	0.06
	B7	3	0.9	1.1	0.97	0.12
	B8	3	0.8	0.9	0.87	0.06
	B9	3	0.9	1.1	1.00	0.10
	Average/mean		0.91	1.18	1.09	

**Table 5:** Association of dental fluorosis with sources of drinking water.

Variables	The Severity of Dental Fluorosis						
	None	Questionable	Very Mild	Mild	Moderate	Severe	Total
Well water	163	141	105	71	12	3	495
Filtered water	414	197	36	5	3	0	655
Total	577	338	141	76	15	3	1150
*p* < 0.002							
